# Millet Bran Dietary Fibers Modified by Heating and Enzymolysis Combined with Carboxymethylation, Acetylation, or Crosslinking: Influences on Properties of Heat-Induced Egg White Protein Gel

**DOI:** 10.3390/foods13172827

**Published:** 2024-09-05

**Authors:** Yan Li, Chen Feng, Xueying Wang, Yajun Zheng, Xinling Song, Nan Wang, Danhong Liu

**Affiliations:** 1 Food Science College, Shanxi Normal University, Taiyuan 030092, China; xssxsd2019@yeah.net (Y.L.); fengchen8098@163.com (C.F.); 13546675098@163.com (X.S.); 18635975320@163.com (N.W.); ldh20041104@163.com (D.L.); 2 College of Food Science, Northeast Agricultural University, Harbin 150030, China; wxy18835739022@163.com

**Keywords:** millet bran dietary fiber, heating, composite modifications, egg white protein gel, texture quality, gastrointestinal digestion, antioxidant activity

## Abstract

Applications of millet bran dietary fiber (MBDF) in the food industry are limited by its poor hydration properties. Herein, MBDF was modified by heating, xylanase and cellulase treatment separately combined with carboxymethylation, acetylation, and phosphate crosslinking, and the effects of the modified MBDFs on heat-induced egg white protein gel (H-EWG) were studied. The results showed that three composite modifications, especially heating and dual enzymolysis combined with carboxymethylation, increased the surface area, soluble fiber content, and hydration properties of MBDF (*p* < 0.05). MBDF and the modified MBDFs all made the microstructure of H-EWG denser and decreased its α-helix content. Three composite modifications, especially heating and dual enzymolysis combined with carboxymethylation, enhanced the improving effect of MBDF on the WRA (from 24.89 to 35.53 g/g), pH, hardness (from 139.93 to 323.20 g), chewiness, and gumminess of H-EWPG, and enhanced the gastric stability at 3–5 g/100 g. MBDFs modified with heating and dual enzymolysis combined with acetylation or crosslinking were more effective in increasing the antioxidant activity of the gastrointestinal hydrolysates of H-EWG than MBDF (*p* < 0.05). Overall, heating, xylanase and cellulase treatment separately combined with carboxymethylation, acetylation and crosslinking can enhance the hydration properties and the improving effect of millet bran fibers on H-EWG properties.

## 1. Introduction

Dietary fiber has various healthcare functionalities and wide applications, such as as a thickener, gelling agent, texture improver, and emulsifier in food, and use in the cosmetic and medical industries, which are positively correlated with its solubility and hydration properties [1]. Dietary fibers with a soluble dietary fiber content of more than 30% are considered to be ideal. The source of dietary fiber can be found in cereal byproducts which are inexpensive and available, but most cereal byproduct dietary fiber contains a low content of soluble dietary fiber (approximately 4 g/100 g) [2]. Millet (*Setaria italica*) bran is the main byproduct of millet processing with a high content of dietary fiber (55 g/100 g) and annual output of about 5000 t in China, but millet bran dietary fiber (MBDF) has low soluble dietary fiber content, relatively poor hydration and gelling properties and limited application in the food industry [3]. Recently, different modifications, including chemical (acidic and alkaline treatments, carboxymethylation, acetylation, and crosslinking) [4,5,6], biological (enzyme hydrolysis and fermentation) [7,8], and physical modifications (heating, extrusion cooking, ultrafine grinding, ultrasonic treatment, and high-pressure homogenization), have been utilized to increase the solubility of dietary fibers [9,10,11]. Zhu et al. [7] demonstrated that cellulase and hemicellulase hydrolysis is capable of degrading molecular chains of MBDF and exposing hydrophilic groups, while Zheng et al. [6] revealed that cellulase hydrolysis combined with chemical modification, including carboxymethylation, acetylation, and crosslinking, can increase the hydration properties of MBDF via grafting more polar groups. A combination of these approaches may be more effective to increase the functional properties of MBDF, though to our best knowledge, data about the effect of heating and enzymolysis separately combined with carboxymethylation, acetylation, and crosslinking has not been reported.

More importantly, it has been shown that the gel characteristics of heat-induced egg white gel (H-EWG) can be improved by the addition of dietary fibers. H-EWG is widely used in food, pharmaceuticals, cosmetics, and organic materials for its unique gelation properties, such as high elasticity, springiness and chewiness, but its hardness, gumminess, water-retention ability, and freeze-thaw properties are relatively lower [12]. Moreover, its high transparency limits the application of EWPG in the preservation of photosensitive food [13]. Dextran sulfate polysaccharide increased the hardness and water-holding ability of H-EWG [14,15], and a mixed gel of carboxymethyl cellulose and ovalbumin had a higher elasticity modulus [16]. Moreover, if dietary fiber is added to H-EWG, it will be conferred with the functional activities of dietary fibers, such as hypolipidemic, hypoglycemic, and antioxidation activities, etc. However, research on the effect of dietary fibers on H-EWG is limited. Therefore, to improve the functionalities of MBDF and expand its applications in hydrogels, MBDF was modified by heating, and cellulase and xylanase hydrolysis separately combined with carboxymethylation, crosslinking, and acetylation. The effects of the composite modifications on the structure and hydrophilicity of MBDF, and the influence of the modified MBDFs on the gel properties of H-EWG were studied. This study will provide new ways to improve the functional characteristics of dietary fibers and egg white gel-based food.

## 2. Materials and Methods

### 2.1. Materials

Nanliu Grain Farm (Wuxiang, China) donated millet bran, which was harvested in August 2023. Egg white protein powder (protein content was above 95%) was purchased from Zhongyi Egg Products Co., Ltd., Suqian City, Anhui Province, China. Cellulase (from Aspergillus niger, 5.0 × 10^5^ U/g), xylanase (from Trichoderma viride G, 1.5 × 10^4^ U/g), α-amylase (from Bacillus licheniformis, 1.0 × 10^5^ U/g), pepsin (from porcine stomach mucosa), and trypsin (from bovine pancreas, 0.25 × 10^3^ U/g) came from the Yigao Chemical Factory, Tianjin City, China. Sodium trimetaphosphate, propylene oxide, and other reagents were analytically pure and purchased from Dinging Reagent Co., Guangzhou, China.

### 2.2. Extraction of MBDF

MBDF was extracted using the same procedures cited in Zheng and Li [17]. Briefly, millet bran was air-dried (45 °C, 3 h), milled with a LC-2TX grinder (Xinglin Mill Instrument Factory, Foshan, China) and deoiled using petroleum ether (boiling point range of 60–90 °C) triplicates. Secondly, the defatted millet bran (5 g/100 mL, suspended in 0.1 mol/L of phosphate buffer) and α-amylase (1:100, g/g, enzyme to millet bran) were incubated at 90 °C, 205 rpm, and pH 5.0 using a BHE-002Z shaker (Suzhou Shaker Factory, Suzhou, China) for 150 min. After that, Alcalase (8: 1000, g/g) was put in and the dispersion was incubated at 50 °C, 205 rpm, and pH 9.0 for 180 min, followed by amyloglucosidase (8:1000, g/g raw material) at 60 °C, 205 rpm, and pH 2.0 for 150 min. Next, these enzymes were inactivated via heating at 100 °C for 10 min. After filtration and washing with deionized water, the residue was air-dried (45 °C, 3 h) to obtain MBDF.

### 2.3. Heating and Dual Enzymes Treatment of MBDF

MBDF (40 g) was subjected to heat treatment (121.3 °C) and high pressure (103.4 kPa) for 45 min using a DZFZ-6C Autoclave (Shandong Boke Instrument Factory, Jinan, China). Afterward, xylanase (60 U/g) and cellulase (40 U/g) were added into the MBDF dispersion (1 g/15 mL), which was shaken at 50 °C, 205 rpm, and pH 5.0 for 150 min [5]. The reaction was terminated via heating treatment (100 °C, 10 min). After cooling and filtration, the residue on the filter paper was air-dried (45 °C, 6 h) to obtain heat-and-dual-enzymes-treated millet bran dietary fiber (MBDF-HDE).

### 2.4. Carboxymethylation of MBDF-HDE

MBDF-HDE (8 g) dispersed into 85% ethanol (*v*/*v*, 80 mL) was magnetically stirred at 215 rpm for 30 min [18]. Simultaneously, sodium hydroxide solution (1.13 mol∙L^−1^, 10 mL) was mixed with 40 mL of ethanol (85%, *v*/*v*) at 215 rpm and 35 °C for 45 min, and then added into the MBDF-HDE dispersion, which was stirred at 215 rpm and 35 °C for 65 min. Next, sodium hydroxide solution (3.38 mol∙L^−1^, 40 mL) and chloroacetic acid (3.38 mol∙L^−1^, 20 mL) were added into the reaction dispersions. After stirring at 215 rpm and 53 °C for 210 min, the reaction mixture was cooled and adjusted to pH 7.0 using acetic acid (50%, *v*/*v*), and then filtered using Whatman 1007-04B paper. The residue was collected and washed in triplicates using anhydrous ethanol (60 mL). After drying at 48 °C for 12 h, MBDF modified by heating, dual enzymatic hydrolysis, and carboxymethylation (MBDF-HDEC) was obtained. The substitution degree of the carboxymethyl group was measured applying procedures from Zhang et al. [19].

### 2.5. Phosphate Crosslinking of MBDF-HDE

MBDF-HDE (10 g), sodium tripolyphosphate (0.24 g) and sodium trimetaphosphate (2.4 g) were dispersed in 100 mL deionized water (dH_2_O), which was adjusted to pH 11.0 with 1 mol∙L^−1^ of NaOH [20]. The reaction was stirred at 300 rpm for 180 min at 45 °C, and then was stopped by being adjusted to pH 7.0 using 1 mol∙L^−1^ of HCl. After filtration, the residue was collected and washed using dH_2_O (80 mL). MBDF modified by heating, dual enzymatic hydrolysis, and phosphate crosslinking (MBDF-HDEPC) was gained after drying at 40 °C overnight.

### 2.6. Acetylation of MBDF-HDE

MBDF-HDE (10 g) was dispersed in dimethyl sulfoxide (75 mL) and magnetically stirred at 300 rpm and 70 °C for 180 min [21]. After being cooled to 45 °C, the reaction solution was mixed with 0.1 g sodium carbonate and 0.716 g/L of isopropenyl acetate (*w*/*v*, 3.35 mL) and then stirred at 45 °C and 300 rpm for 72 h. After being filtered with Whatman 1007-04B paper, the residue was collected and washed using isopropyl alcohol (60 mL). After air-drying at 48 °C for 12 h, MBDF modified by heating, dual enzymatic hydrolysis, and acetylation (MBDF-HDEA) was achieved. The acetylation degree was measured applying procedures from Nasseri et al. [21].

### 2.7. Preparation of Egg White Protein Gels with MBDFs

Fifteen grams of egg white powder was dissolved in 100 mL dH_2_O and then kept at 4 °C overnight [14]. An amount of 20 mL of egg white solution was transferred into a 50 mL beaker, and then MBDF, MBDF-HDEC, MBDF-HDEA, and MBDF-HDEPC in various amounts (1–5 g/100 g) were added, respectively. After 3 min of stirring at 90 °C, the mixture in the beaker was set aside at 90 °C for 27 min. Afterward, the mixture was cooled by running tap water and kept at 4 °C for 7 h to obtain heat-induced egg white gel (H-EWG), and heat-induced egg white gels separately fortified with MBDF (H-EWG/MBDF), MBDF-HDEC (H-EWG/MBDF-HDEC), MBDF-HDEPC (H-EWG/MBDF-HDEPC), and MBDF-HDEA (H-EWG/MBDF-HDEA).

### 2.8. Chemical Constituents, Surface Area, and Color Determination

The soluble, insoluble, and total dietary fiber contents of MBDF, MBDF-HDEH, and MBDF-HDEAG were determined by the AOAC.991.43 method [22]. The contents of protein, moisture, ash, and fat were determined using methods AOAC.955.04, AOAC.920.39, AOAC.924.05, and AOAC.92.05, respectively [22]. The acid insoluble lignin, acid, and neural detergent fiber contents of the MBDFs were determined to calculate the lignin, hemicellulose, and cellulose contents, respectively. Particle size expressed as D_3,2_ (Sauter mean diameter, μm) and surface area (m^2^/kg) of MBDFs were analyzed using a JFNER-LB particle size analyzer (Jingbei Frontier Instrument Co., Chengdu, China). The polydispersity index was calculated by comparing the Dx (90), Dx (10), and Dx (50) values (representing the particle diameters of 10%, 50%, and 90% of the sample, respectively) [17]. Additionally, color indexes, including *L* (indicative of the lightness), *b* (representative of the redness), and *a* (corresponding to the yellowness) of the MBDFs, were measured with an NH130 High Quality Portable Color Difference Meter (Three-NH Colorimeter Co., Shenzhen, China). Then, the color difference (Δ*E*) across MBDF and the modified MBDFs was calculated as follows:

(1) $$∆E=\sqrt{{\left(L-L_{0}\right)}^{2}+{\left(a-a_{0}\right)}^{2}+{\left(b-b_{0}\right)}^{2}}$$

where *L*_0_, *a*_0_, and *b*_0_ are the lightness, redness, and yellowness of untreated MBDF, respectively.

### 2.9. Structure Investigation

#### 2.9.1. Microstructure Microscopy

First, MBDFs and H-EWPGs were coated with a 10 nm gold layer. Then, the samples were scanned by a scanning electron microscopy (JOLE-JMS-5700E, Tokyo, Japan). The accelerating voltage, scale bar, and magnification were 10 kV, 1 μm, and 5000, respectively [17].

#### 2.9.2. Fourier-Transformed Infrared Spectroscopy

The MBDFs and H-EWGs were freeze-dried in a vacuum freeze-dryer (HFD-6, Heyuan Ice Equipment Co., LTD, Zhengzhou, China), mixed with dry KBr to form pallets (with a thickness of 10 nm), and then were scanned by a Fourier-transformed infrared (FT-IR) spectrometer (8400S, Shimadzu, Japan). PeakFit software v4.12 (Seasolve, Framingham, MA, USA) was employed to investigate the secondary structure of H-EWPGs applying the procedure from Bashash et al. [23] with wavenumber ranging from 4000 to 400 cm^−1^.

### 2.10. Water-Retention and Expansion Abilities and Viscosity

The water-expansion ability (WEA) and retention ability (WRA) of samples were determined applying the same procedures from Zheng and Li [17]. The viscosity of MBDFs was measured applying the procedure of Zhu et al. [7] by a RAV-I5 viscometer (Shengyebao Niandu Instrument Factory, Guangzhou, China).

### 2.11. Characterization of Gel

#### 2.11.1. Water-Retention Ability of Gel

The centrifugation method was adopted to determine the water-retention ability (WRA) of the gel samples [24]. Briefly, in a centrifuge tube (50 mL), approximately 2 g (M_0_) of H-EWGs was added along with filter paper. After filtration at 4000× *g* at 4 °C for 20 min, the gel samples were weighed again (*M*_1_). WRA (%) was calculated according to the following equation:

(2) $$WRA\left(\%\right)=M_{1}/M_{0}\times 100\%\;$$

where *M*_0_ and *M*_1_ represent the gel weight before and after centrifugation, respectively. 

#### 2.11.2. Freeze-Thaw Cycle (FTC) Test

The gel samples were loaded in a 15 mL glass centrifuge tube for syneresis measurement [24]. The freeze-thaw cycle contained two steps: (i) the gels in the tube were frozen at −16 °C for 24 h; and (ii) the frozen gels were slowly thawed for 8 h at room temperature (approximately 25 °C). The gel went through five of these cycles and then weighed (*W_s_*). After filtration (3500*× g*, 15 min), the weight of the residue was measured and recorded as *W_d_*. The dehydration rate in the freeze-thaw cycle was quantified according to Equation (3):

(3) $$Dehydrationrate\left(\%\right)=(W_{s}-W_{d})/W_{s}\times 100\%\;$$

#### 2.11.3. Optical Transparency of Gel

The gel sample was transferred to a quartz colorimetric dish, and the absorbance was determined by a JH754PC UV-Vis spectrophotometer (Shanghai Jinghua Co., Ltd., Shanghai, China) at 600 nm [14].

#### 2.11.4. Texture Properties of Gel

The textural properties of H-EWGs, including hardness, elasticity, cohesiveness, adhesiveness, chewability, and resilience were tested using a TA Plus Texture Analyzer System (LLOYD Instrument Co., Hong Kong, China) with a P/36 R probe. The data were measured in TPA mode, and the test parameters were as follows: the trigger force, compression rate, and the test, pre-test, and post-test speeds were 3 g, 50%, 1, 2, and 1 mm/s, respectively [15].

#### 2.11.5. In Vitro Gastrointestinal Digestion

The in vitro gastrointestinal digestion of H-EWPGs was determined applying the procedures from Lee et al. [25]. The simulated gastric fluid (pH 2.0) was prepared by mixing NaCl (2 g/L), pepsin (4000 U/mL), and HCl (0.1 mol/L), and the intestine digest fluid (pH 7.0) consisted of trypsin (800 U/mL), bile salt (1 g/100 mL), NaCl (0.8775 g/100 mL), and K_2_HPO_4_ (0.68 g/100 mL). H-EWPGs (4 g) were smashed and mixed with 20 mL of the simulated gastric fluid in a conical flask, and then shaken at 120 rpm and 37 °C for 1 h to simulate the gastric digestion stage. Next, the mixture was modulated to pH 7.0, and then 20 mL of the simulated intestine fluid was added, and then shaken at 160 rpm and 37 °C for 2 h. During the whole digestion, equivalent digestive juice (1 mL) was taken off per 0.5 h for free amino acid content determination, and the reaction solution was kept at the same volume by adding the corresponding digestive fluid. The o-phthalaldehyde (OPA) assay was adopted to quantify the free amino acid content of the transferred digestive juice [26]. The OPA reagent contained sodium dodecyl sulfate (10 g/100 mL, 5 mL), β-mercaptoethanol (200 μL), sodium tetraborate (50 mmol/L, 5 mL), and o-phthalaldehyde (20 mg/1 mL methanol). The reaction solution (digestive juice: OPA reagent = 1:20, *v*/*v*) was incubated at 37 °C for 2 min, and then a JH754PC spectrophotometer (Shanghai Jinghua Co., Ltd., Shanghai, China) was employed to determine the absorbance at 340 nm. Additionally, the scavenging capacity of the gastrointestinal hydrolysates on ABTS radicals was measured citing the procedures from Garzón et al. [27].

### 2.12. Statistical Analysis

Each determination was repeated for more than three times. V.17.0 SPSS software (IBM Co., Chicago, IL, USA) was employed to analysis the significant differences across data with analysis of variance (ANOVA) as well as Duncan’s multiple comparisons at a significance level of *p* < 0.05 [16]. OriginPro 2021b SR1 v9.8.5.204 (OriginLab Com., Northampton, MA, USA) with a license was used for the graphical representations. 

## 3. Results and Discussion

### 3.1. Chemical Constituent of MBDFs

As shown in Table 1, insoluble dietary fiber (IDF) was the dominant component of MBDF, with hemicellulose being the main component (57.00 g/100 g), consistent with the results of Chu et al. [28]. There was no significant difference in the fat and protein contents across the MBDFs (*p* > 0.05), but the ash content of MBDF-HDEC and MBDF-HDEA was higher (*p* < 0.05), perhaps attributable to the addition of salts or alkali during acetylation and carboxymethylation. After the three composite modifications (heating and dual enzymolysis separately united with phosphate crosslinking, acetylation, and carboxymethylation), the IDF contents of MBDF-HDEC, MBDF-HDEA, and MBDF-HDEPC were all decreased (*p* < 0.05), ascribed to the degradation of hemicellulose and cellulose by the dual enzymes, and the introduction of more hydrophilic groups, including carboxymethyl, phosphate, and acetyl groups. High pressure and heating (121.3 °C, 0.11 MPa) can split polysaccharide chains of DFs and some hydrophilic groups were released as a consequence [7]. Correspondingly, a higher (*p* < 0.05) SDF content was observed in MBDF-HDEC, MBDF-HDEPC, and MBDF-HDEA, revealing that heating and dual-enzymolysis separately united with acetylation, phosphate crosslinking, and carboxymethylation effectively induced the conversion of insoluble fiber to soluble fiber. Moreover, MBDF-HDEC showed a high SDF content, which was approximately 2.2 times that of heating and cellulase-hydrolyzed MBDF (2.47 g/100 g) [5] and carboxymethylated MBDF (2.51 g/100 g) [6], revealing that a combination of heating, dual enzymolysis, and carboxymethylation was more effective to increase the hydrophilicity of MBDF than any single treatment. Additionally, a higher SDF content was observed for MBDF-HDEC in comparison to MBDF-HDEPC and MBDF-HDEA (*p* < 0.05), resulting from the introduced carboxymethyl group, which had greater polarity than the phosphate and acetyl groups [29]. 

### 3.2. Particle Size and Color Analysis of MBDF

DFs of large size will create an unacceptable impact on the sensory quality and taste of egg white gel [12]. The specific surface area of MBDF was increased but its particle size (D_3,2_) was decreased after the mixed modifications (Table 1), which probably accounted for the degradation of the fiber chains resulting from heating, dual enzymatic hydrolysis, carboxymethylation, acetylation, or crosslinking [6]. MBDF-HDEA had the largest surface area (3226.70 cm^2^/cm^3^), consistent with its smallest particle size. An increase in the surface area of DF may enhance its interactions with water or oil molecules [18]. The polydispersity index indicates the uniformity of the particle size distribution [17]. MBDF-HDEA exhibited a lower polydispersity index than MBDF, suggesting that heating and dual enzymolysis combined with acetylation made the particle size distribution of MBDF more uniform. 

Color is an important factor that affects DF’s application as well as consumer choice. Compared with MBDF, MBDF-HDEC, MBDF-HDEPC, and MBDF-HDEA, all showed considerable color difference (∆*E*) with decreased *L* values (representative of lightness), but increased *a* or *b* values (indicative of redness and yellowness, respectively) (*p* < 0.05), which might be due to the degradation of natural pigments under heating and a browning reaction occurring during the chemical modification process [29]. MBDF-HDEPC and MBDF-HDEA showed higher ∆*E* than MBDF-HDEC, indicating that phosphate crosslinking and acetylation had a negative impact on the brightness of MBDF. Previous studies also found that thermal or chemical treatment lowered the brightness of dietary fibers [5,10,17]. 

### 3.3. Structure Analysis of MBDF

#### 3.3.1. Microstructure of MBDF

**Figure 1:**
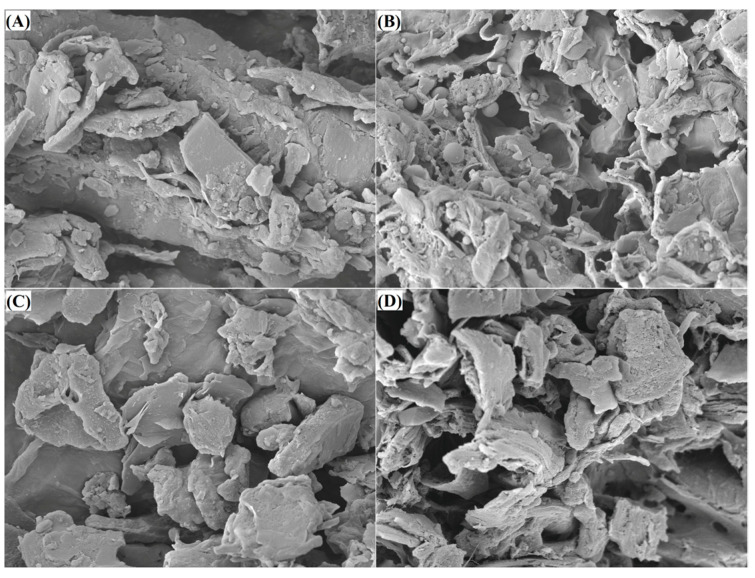
Scanning electron micrographs of MBDF (**A**), MBDF-HDEC (**B**), MBDF-HDEPC (**C**), and MBDF-HDEA (**D**) with a magnification of 5000×, at 1 μm. MBDF, millet bran dietary fiber; MBDF-HDEC, MBDF modified by heating and dual enzymes hydrolysis combined with carboxymethylation; MBDF-HDEPC, MBDF modified by heating and dual enzymes hydrolysis combined with phosphate crosslinking; MBDF-HDEA, MBDF modified by heating and dual enzymes hydrolysis combined with acetylation.

Figure 1A–D show the changes that occurred in the microstructure of MBDF after the three mixed modifications. MBDF showed a relatively smooth surface microstructure with a small amount of porosity and debris (Figure 1A). By contrast, a distinct honeycomb microstructure with more porosity or debris was observed in MBDF-HEC, MBDF-HEPC, and MBDF-HEA (Figure 1B–D). The increase in porosity or debris mainly resulted from the breakdown of glycosidic linkages induced by heating, dual enzymatic hydrolysis, and alkali treatment during the chemical modifications [19,29]. Moreover, the increase in holes and fragments on the microstructure can enhance the interactions of fibers with water or other molecules, potentially leading to differences in the gel properties [24].

#### 3.3.2. Fourier-Transform Infrared Spectra

**Figure 2:**
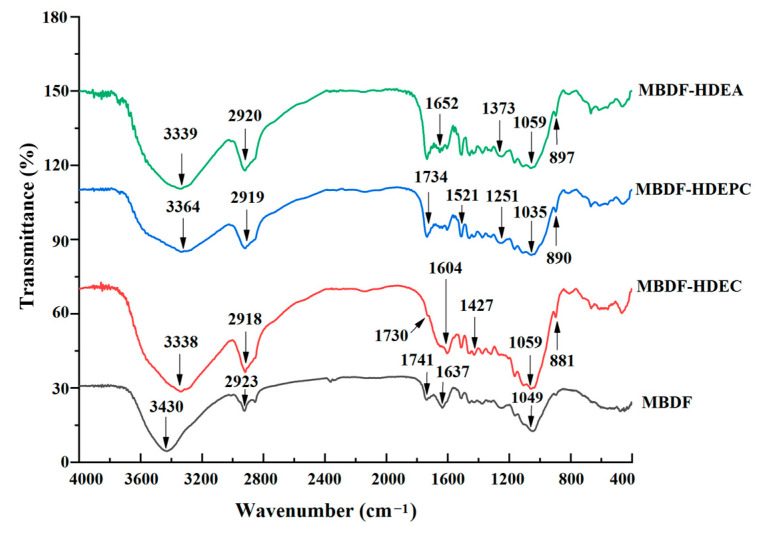
Fourier-transformed infrared spectroscopy of MBDF, MBDF-HDEC, MBDF-HDEPC, and MBDF-HDEA.

As presented in Figure 2, the untreated and modified MBDFs all had strong peaks at approximately 3420, 2920, 1650, and 1050 cm^−1^, respectively, conforming to the typical FT-IR spectra of dietary fibers. Moreover, changes in several characteristic peaks revealed that heating, and dual enzymolysis united with acetylation, crosslinking, or carboxymethylation affected the MBDF structure, especially the chemical bonds and functional groups. The peak at 3430 cm^−1^ in the spectrum of MBDF, which represented the asymmetric stretching of O-H, separately transferred to 3338, 3364, and 3339 cm^−1^ in the spectra of MBDF-HDEC, MBDF-HDEPC, and MBDF-HDEA, indicating that the synthesis modifications changed the hydrogen bonds in MBDF [9]. The spectra of MBDF-HDEC, MBDF-HDEPC, and MBDF-HDEA all had a new absorption peak near 890 cm^−1^ (indicating asymmetric stretching of β-C-H), resulting from the breakdown of β-glycosidic linkages caused by heating and/or dual enzymes hydrolysis [8]. In the spectrum of MBDF-HDEPC, adsorption peaks in 1251 cm^−1^ (indicating the deformation of the P=O bond) and 1521 cm^−1^ (representing the vibration of C-H) confirmed successful grafting of phosphate groups on MBDF [8]. In the spectrum of MBDF-HDEC, the peaks appeared at 1730 and 1427 cm^−1^, suggesting that carboxymethyl groups had been grafted on the MBDF [19]. Moreover, across the spectra of MBDF and MBDF-HDEA, the red-shift that was observed on the peaks at 1652 and 2920 cm^−1^ showed that acetyl groups had been introduced into the MBDF molecules [20]. Similar results were obtained by previous studies [5,17]. 

### 3.4. Hydration Properties of DF

Hydration characteristics including the water-retention and expansion abilities, and the viscosity of DFs are positively related with their gel properties [16]. All MBDF-HDEC, MBDF-HDEPC, and MBDF-HDEA had higher (*p* < 0.05) water-holding and expansion abilities than MBDF (Table 1). The improvement in WHA and WSA of MBDF after heating and dual enzymolysis separately united with acetylation, carboxymethylation, and crosslinking predominately occurred through two ways: (1) making the microstructure of MBDF more porous and fragmented (Figure 1) and increasing its surface area (Table 1) to enhance the affinity between water and MBDF; (2) grafting acetyl, carboxymethyl, and phosphate groups on MBDF to increase its hydrophilicity [17,18]. Moreover, the reasons for the high WHA and WSA of MBDF-HEPC also included the network structure between the fiber chains formed after phosphate crosslinking, which can retain more water molecules [20]. MBDF-HDEC exhibited the highest WHA and WSA, followed by MBDF-HDEA and MBDF-HDEPC, which was consistent with the order of their SDF contents (Table 1).

Polysaccharides with high viscosity are considered to have a greater ability to form gels or improve the gel properties of egg white protein [15]. Both MBDF-HDEC and MBDF-HDEPC exhibited higher (*p* < 0.05) viscosity than MBDF (Table 1), corresponding to their larger surface area, higher WHA, SDF content, and WSA, as well their more porous or multichip microstructures (Table 1, and Figure 1B,C). Improvement of the SDF content means that more fibers can contribute to the viscosity of the aqueous solution; meanwhile, increase in the WHA, WSA, and surface area can enhance the interactions of fibers with water molecules, which can all increase the viscosity of MBDF [30].

It is worth mentioning that the WHA, WSA, and viscosity of MBDF-HDEC and MBDF-HDEPC were higher than MBDF treated by dual enzymolysis separately united with carboxymethylation (2.65 g/g, 0.81 mL/g, and 13.77 cP) and phosphate crosslinking (2.25 g/g, 0.49 mL/g, and 14.67 cP) [6], highlighting that heating has an obvious improvement effect on the hydration properties of MBDF. Additionally, the high hydration properties indicate the potential applications of MBDF-HDEC, MBDF-HDEPC, and MBDF-HDEA in the food industry as water-retaining agents and texture improvers [1]. 

### 3.5. Structural Characteristics of H-EWGs

#### 3.5.1. Microstructure of Gels

**Figure 3:**
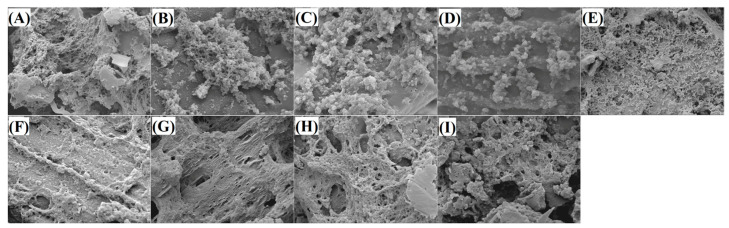
Scanning electron micrographs of native egg white gel (**A**), egg white gel containing 5 g/100 g MBDF (**B**), egg white gel containing 5 g/100 g MBDF-HDEPC (**C**), egg white gel containing 5 g/100 g MBDF-HDEA (**D**); egg white gel containing 5 g/100 g MBDF-HDEC (**E**), egg white gel containing 4 g/100 g MBDF-HDEC (**F**), egg white gel containing 3 g/100 g MBDF-HDEC (**G**), egg white gel containing 2 g/100 g MBDF-HDEC (**H**), and egg white gel containing 1 g/100 g MBDF-HDEC (**I**) with a magnification of 5000×, at 1 μm.

The scanning electron micrographs of H-EWG and H-EWGs fortified with MBDF (H-EWG/MBDF), MBDF-HDEPC (H-EWG/MBDF-HDEPC), MBDF-HDEA (H-EWG/MBDF-HDEA), and MBDF-HDEC (H-EWG/MBDF-HDEC) at an addition amount of 5 g/100 g are shown in Figure 3A–E, respectively. H-EWG had a smooth surface and relatively rough inner microstructure with many pores (Figure 3A); H-EWG/MBDF, H-EWG/MBDF-HDEPC and H-EWG/MBDF-HDEA all showed a more dense and granular microstructure (Figure 3B–D); meanwhile H-EWG/MBDF-HDEC had a relatively denser thread-like microstructure with a large amount of tiny holes (Figure 3E). During preparation of heat-induced hydrogels, the addition of fibers can provide a fiber skeleton on which egg white protein aggregates, which is helpful to the formation of a dense and granular three-dimensional microstructure with tiny holes [12]. Moreover, the skeleton structure of the fiber matrix can enhance the interactions between egg white proteins and promote their adsorption on the surface of gels, leading to a rough but dense granular microstructure [31,32]. In comparison with the microstructure of H-EWG/MBDF (Figure 3B), the microstructures of H-EWG/MBDF-HDEPC and H-EWG/MBDF-HDEA were more granular and denser, mainly due to the grating of acetyl and phosphate groups, which can improve the crosslinking between egg white proteins [33].

The microstructures of H-EWGs with MBDF-HDEC at addition amounts of 4, 3, 2 and 1 g/100 g are shown in Figure 3F–I, respectively. It is obvious that the microstructure of H-EWG became more granular and denser, and the holes became smaller as the addition amount increased, demonstrating that increasing the addition amount of MBDF-HDEC can enhance the interaction between egg white proteins and promote their aggregation. However, the microstructure of H-EWG/MBDF-HDEC seems to be more delicate at an addition amount of 4 g/100 g (Figure 3F) than that at 5 g/100 g (Figure 3E); therefore, an excessive addition amount of DF is not conducive to the structure of H-EWG. Chu et al. [28] found that addition of too much fiber made the gels taste rough, too.

#### 3.5.2. Secondary Structure

**Figure 4:**
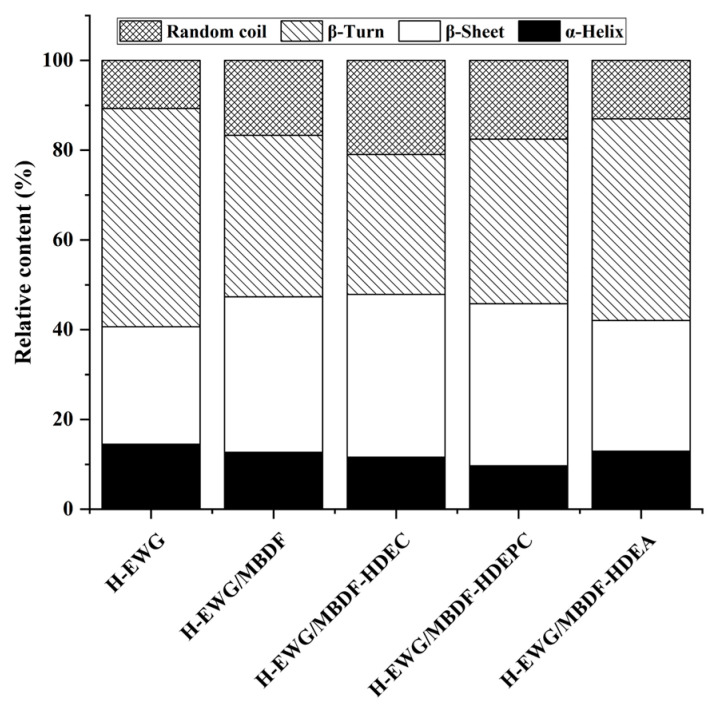
Relative content of protein secondary structure of heat-induced egg white gels fortified with MBDFs at an addition amount of 5 g/100 g. H-EWP, heat-induced egg white protein gel; H-EWP/MBDF, heat-induced egg white gel with MBDF; H-EWP/HDEC, heat-induced egg white gel with MBDF-HDEC; H-EWP/HDEPC, heat-induced egg white gel with MBDF-HDEPC; and H-EWP/HDEA, heat-induced egg white gel with MBDF-HDEA.

It was obvious that the α-helix and β-turn contents of H-EWG were significantly decreased (*p* < 0.05), but its random coil and β-sheet contents were increased after the addition of MBDF, MBDF-HDEC, and MBDF-HDEPC (Figure 4). The higher random coil content and lower α-helix content indicated that the structure of the egg white protein became more stretched [32], which was conducive to the formation of a more dense and granular microstructure (Figure 3C,E). During heat-induced gelation, MBDFs with considerable water-retention and expansion abilities can increase the interactions between water and egg white proteins, and make the proteins’ structure more stretched by increasing the intermolecular steric hindrance, resulting in an increase in random coil and a decrease in α-helix [30]. The hydrogen bond is one of the main forces maintaining the β-sheet of proteins. The carboxymethyl group of MBDF-HDEC and the phosphate group of MBDF-HDEPC were both helpful to the formation of hydrogen bonds across the egg white proteins, facilitating the formation of β-sheet in H-EWG [34]. There was no remarkable difference in secondary structure between H-EWG and H-EWG/MBDF-HDEA (*p* > 0.05), perhaps because the introduced acetyl group had a low polarity [21]. Xu et al. [30] also verified that the addition of dietary fibers increased the random coil content of egg white protein gel. 

### 3.6. Physicochemical Properties of H-EWGs

#### 3.6.1. WHA of H-EWGs

**Figure 5:**
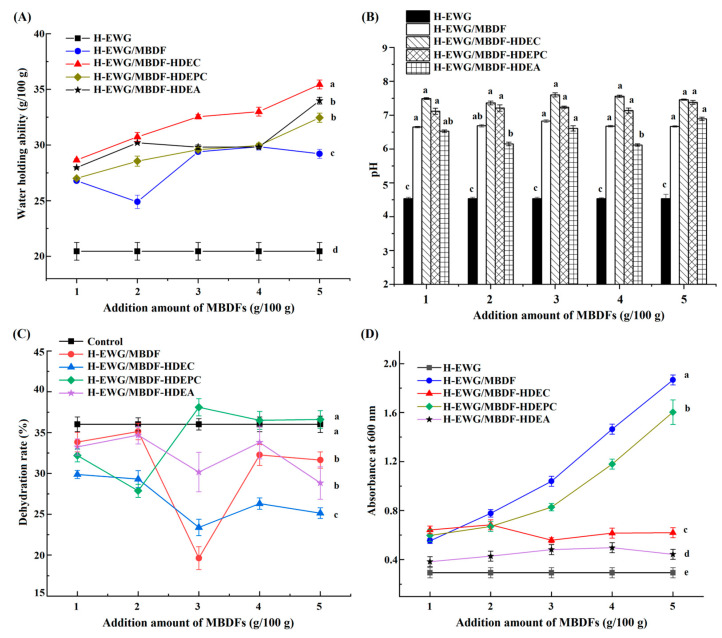
Effects of different addition amount of MBDFs on the water-holding ability (**A**), pH (**B**), dehydration rate in the freeze-thaw cycle (**C**), and optical transparency (**D**) of heat-induced egg white gel (H-EWG). Different lower letters (a–e) near the lines or on the bars mean significant difference (*p* < 0.05).

Figure 5A shows that the WHA of H-EWG was increased by addition of MBDF, MBDF-HDEC, MBDF-HDEPC, or MBDF-HDEA (*p* < 0.05), and that their improvement effects are dose-dependent. One reason is that these MBDFs all have considerable WHA (1.79–3.07 g/g, Table 1), which can enhance the affinity of egg white proteins with water [14]. Another reason is that addition of these MBDFs increased the random coil content of H-EWG (Figure 4) and the number of tiny holes in the microstructure (Figure 3B–E), which were both helpful to the unfolding of the gel structure and the interactions between H-EWG and water molecules [31]. Furthermore, MBDF-HDEC exhibited the highest ability to improve the WHA of H-EWG at an addition amount of 5 g/100 g, mainly attributable to its highest SDF content and WHA (Table 1), and the granular microstructure with many tiny holes (Figure 3E). Previous studies also showed that the WHA of protein gels can be increased by dietary fibers [30,34]. The high water holding capacity indicates that egg white protein gels fortified with MBDFs can be used as antagonists, water retaining film, or hydrogels [2]. 

#### 3.6.2. pH Value

Changes in the pH value can affect the formation mechanism, texture quality, and shelf-life of hydrogels [35]. As shown in Figure 5B, the pH value of H-EWG was approximately 4.5, which is near the isoelectric point of ovalbumin (the predominant constituent of egg white protein) [36]. The pH value of H-EWG increased from 4.5 to 6.12–7.60 after the addition of MBDFs with an addition amount of 1–5 g/100 g (*p* < 0.05). At the isoelectric point, the interactions between egg white proteins were low and the gels formed had low hardness, water-retarding capacity, and transparency [12]. A dense gel with a continuous network structure can be formed between the egg white proteins at around pH 7.0 [24], which is one reason why the addition of MBDFs made the microstructure of H-EWG more dense (Figure 3B–E). Moreover, the pH value of H-EWG/MBDF-HDEA was lower than that of H-EWG/MBDF-HDEC at an addition amount of 2–5 g/100 g (*p* < 0.05), mainly because the introduced acetyl group had stronger acidic dissociation than the phosphate group, or because of the different pH conditions during their respective preparations [29]. Previous studies found that the addition of DF increased the pH value of H-EWG, too [16,25].

#### 3.6.3. Dehydration Rate in Freeze-Thaw Cycle

Inhibiting the dehydration of hydrogels in the freeze-thaw cycle can prevent hardening and shrinkage of hydrogel-based foods [23]. The addition of MBDF, MBDF-HDEC, or MBDF-HDEA decreased the dehydration rate of H-EWG (*p* < 0.05, Figure 5C), ascribed to their improvement effects on the microstructure and WHA of H-EWG (Figure 3B,C,E, and Figure 5A), and their reduction effects on the random coil and β-sheet contents of H-EWG (Figure 4), leading to a stronger affinity between the gel and water [37], thus reducing the dehydration rate. Moreover, at 1–3 g/100 g, the dehydration rate of H-EWG decreased as the addition amount of MBDF-HDEC, MBDF, or MBDF-HDEA increased, but at 4–5 g/100 g, the dehydration rate of H-EWG increased as the addition amount increased. The main reason was that the aggregation of egg white proteins was enhanced and the microstructure of H-EWG became denser with increasing addition amount of MBDFs, leading to a decrease in the interactions between the gels and water molecules [25]. Furthermore, H-EWG/MBDF showed the lowest dehydration rate, whereas the addition of MBDF-HDEPC did not change the dehydration rate of H-EWG at 3–5 g/100 g (*p* > 0.05). The phosphate groups in MBDF-HDEPC can improve the egg white proteins’ aggregation and made the microstructure of EWPG became denser (Figure 3C), which was not conducive to water loss in the freeze-thaw cycle [25]. In general, addition of MBDFs at 2–5 g/100 g reduced the dehydration rate of H-EWG, indicating that they can inhibit the deterioration of hydrogel-based food after the freeze-thaw cycle [14]. Ullah et al. [31] verified that okara fiber reduced the freeze-thaw dehydration of tofu gel. 

#### 3.6.4. Optical Transparency of H-EWGs

Optical transparency can influence the applications of gels, and it is dependent on the formation mechanism, the gel structure, the concentration of hydrophilic polymers, pH, temperature, and other factors [25]. The results in Figure 5D revealed that H-EWG with MBDF, MBDF-HDEA, MBDF-HDEPC, or MBDF-HDEC had higher absorbance at 600 nm (*p* < 0.05), showing that addition of these MBDFs lowered the transparency, which was perhaps due to their effects on protein aggregation, microstructure, and the pH of H-EWG. As shown in Figure 5B, the pH of H-EWG was 4.5 (near the isoelectric point of egg white protein), the repulsive force between proteins was weak, and the network structure of gel formed was loose. After the addition of MBDFs, the pH and WHA of H-EWG were both increased (Figure 5A–B), and the interaction between egg white proteins was increased, thus forming a denser and tighter network structure (Figure 3B–E), resulting in higher scattering of light and lower transparency [38]. Moreover, the hydrophobic force, which is a main force in the formation and maintenance of heat-induced gels, can be reduced by the addition of MBDFs, which is helpful in decreasing the transparency of gels [31]. Additionally, MBDF showed the greatest lowering effect on the transparency of H-EWG, followed by MBDF-HDEPC, and their improving effects were dose-dependent, predominately ascribed to MBDF and MBDF-HDEPC promoting the extensive aggregation of egg white proteins and the formation of a denser gel network as the addition amount increased [37]. Apart from that, the phosphate groups in MBDF-HDEPC can promote the crosslinking between egg white proteins and the formation of a molecularly homogeneous network [6], which is helpful in increasing light scattering and reducing transparency. The reduction in the optical transparency means that the use of H-EWG in inhibiting photooxidation can be improved [32].

#### 3.6.5. Texture Properties of H-EWG

The addition of MBDF, MBDF-HDEC, MBDF-HDEPC, and MBDF-HDEA all enhanced the hardness of H-EWG (*p* < 0.05) and showed a positive dose-dependent trend (Table 2). During the formation of the heat-induced gel, MBDFs provide carbon skeletons on which proteins are adsorbed and aggregated, which can enhance the crosslinking of proteins and the gel network strength, resulting in a higher hardness [12]. Moreover, the more granular and denser microstructures of H-EWG/MBDFs (Figure 3B–E) with higher water-holding capacity also enhanced the hardness of gels [31]. The greatest hardness (323.69 g) was observed in H-EWG/MBDF-HDEC, followed by H-EWG/MBDF-HDEPC (272.20 g), which was mainly attributable to their high WHA (Figure 5A). Moreover, the phosphate group of MBDF-HDEPC and the carboxymethyl group of MBDF-HDEPC can both enhance the crosslinking between hydrophilic polymers and thus increase the hardness of H-EWG [29]. 

The gumminess of H-EWG increased with increasing addition amount of MBDFs (Table 2). Gel gumminess represents the energy needed for semi-solid food to become stable, and it is positively correlated with their hardness, cohesiveness, and viscosity [32]. It can be speculated that the increased hardness is one reason for the higher gumminess of H-EWGs with MBDFs. Moreover, the higher β-sheet content (Figure 4) is another reason for the high gumminess of H-EWG/MBDF, H-EWG/MBDF-HDEC, and H-EWG/MBDF-HDEPC [14]. The addition of MBDF-HDEC showed the highest improvement effect on the gumminess of H-EWG across the MBDFs, probably attributable to its highest viscosity (20.00 cP, Table 1). Previous studies evidenced that the addition of sulfate polysaccharide or okara dietary fiber improved the gumminess and hardness of H-EWG [15,31]. 

The chewiness of gels is dependent on gumminess and springiness [30]. The increased gumminess was mainly responsible for the improvement in chewiness of H-EWG after the addition of MBDFs. The increase in pH (Figure 5B) and WHA of H-EWG (Table 1) were both helpful for the formation of a three-dimensional structure with higher chewiness [33]. MBDF-HDEC showed the highest enhancing effect on the chewiness of H-EWG, which is in agreement with its highest viscosity, WHA (Table 1), and the improvement effect on the hardness of H-EWG (Table 2). Increase in hardness, chewiness, and gumminess means that the applications of H-EWG in food, biomedicine, and cosmetics industries will be expanded [35].

By contrast, the springiness and cohesiveness of H-EWG were both reduced (*p* < 0.05) by the addition of MBDF (at 4 g/100 g), MBDF-HDEC (at 2–5 g/100 g), or MBDF-HDEPC (at 5 g/100 g). It was found that DFs can increase the strength of the gel’s network structure via providing carbon skeletons and/or enhancing the WHA of gels, but reduce their springiness as a consequence [12,37]. Moreover, the decreased α-helix and β-turn contents in H-EWG after the addition of MBDFs (Figure 4) were also responsible for the decrease in the springiness of H-EWG. Protein gels with a high content of α-helix have a dense network structure and high springiness [30]. Cohesiveness is indicative of the tensile strength of gels. After the addition of MBDFs, the hydrophobic force between protein molecules was reduced [16], and the pH value, WHA, and hardness of H-EWG were all increased (Figure 5A, Table 1 and Table 2), which were disadvantageous to the tight aggregation of proteins [38], resulting in a lower cohesiveness. Additionally, the addition of these MBDFs did not change the resilience of H-EWG (*p* > 0.05).

### 3.7. In Vitro Digestion of H-EWGs

**Figure 6:**
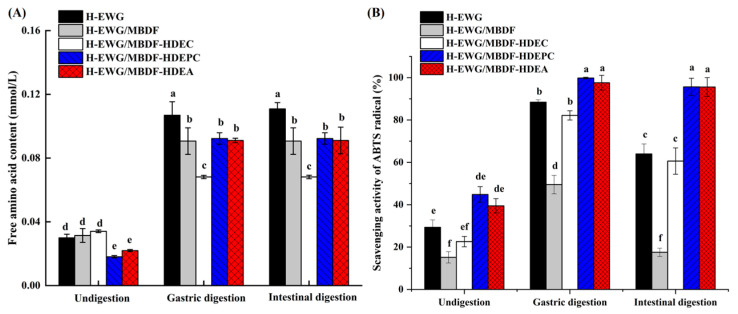
Effects of MBDF, MBDF-HDEC, MBDF-HDEPC, and MBDF-HDEA (at addition amount of 5/100 g) on the free amino acid production (**A**) and ABTS^+^ scavenging activity (**B**) of heat-induced egg white protein gel under gastrointestinal digestion. Different lowercase letters (a–f) in the same column indicate significant difference (*p* < 0.05).

The simulated gastrointestinal digestion of H-EWGs with MBDFs at 5 g/100 g is shown in Figure 6, where the free amino acid content represents the digestion degree. As shown in Figure 6A, significant declining trends were observed in the gastric digestion of H-EWGs with MBDF, MBDF-HDEC, MBDF-HDEPC, or MBDF-HDEA (*p* < 0.05). Except for MBDF-HDEPC, these MBDFs had no obvious effect on the intestinal digestion of H-EWGs (*p* > 0.05), indicating that MBDF, MBDF-HDEC, and MBDF-HDEA improved its stability against gastric digestion but did not affect its bioaccessibility in the small intestine. During gastric digestion, the network structure of H-EWG was destroyed, and egg white protein was released and then hydrolyzed by pepsin to produce peptides and free amino acids [32]. Since dietary fiber cannot be hydrolyzed by pepsin, the addition of MBDFs can inhibit the gastric digestion of H-EWG [1]. The addition of MBDFs increased the pH (Figure 5B), hardness, chewiness, and gumminess of H-EWG, thus increasing its stability against gastric hydrolysis. Moreover, MBDFs can limit the contact of pepsin with the enzymolysis sites of the egg white polypeptide chains, and thus slow down the gastric digestion speed [33]. MBDF-HDEC showed a lower reducing effect on the gastric digestion of H-EWG than MBDF-HDEA (*p* < 0.05), perhaps attributable to the lower interactions between MBDF-HDEC and pepsin [6]. The reducing effect of MBDF-HDEPC on the intestinal digestion of H-EWG may be ascribed to the highest hardness of H-EWG/MBDF-HDEPC (Table 2). Moreover, the phosphate group of MBDF-HDEPC can increase the crosslinking between egg white proteins [29], and thus decrease the intestinal digestion of H-EWG. Liu et al. [14] reported that dextran sulfate addition retarded the digestibility of egg white protein.

As shown in Figure 6B, the addition of MBDF-HDEPC and MBDF-HDEA both increased the ABTS radical scavenging activity of the gastric and intestinal hydrolysates of H-EWG; however, the addition of MBDF decreased the ABTS radical scavenging activity of gastric and intestinal hydrolysates of H-EWG (*p* < 0.05), which seems to contradict the results shown in Figure 6A. Previous studies found that protein hydrolysates may offer higher antioxidant activity as the hydrolysis degree increases [25]; therefore, decrease in digestion of H-EWG should cause a reduction in ABTS radical scavenging activity. On the other hand, free hydroxyl, phenolic acid, and acetyl groups existing in dietary fibers can improve the free radical scavenging activity of gels [27]. Zheng et al. [6] found that crosslinking and acetylation both improved the combined polyphenols content and antioxidant activity of MBDF, thus the addition of MBDF-HDEPC and MBDF-HDEC increased the ABTS radical scavenging activity of H-EWG. The higher antioxidant activity of H-EWG fortified with MBDFs indicates its application in food preservation [24]. 

## 4. Conclusions

MBDF modified by heating, cellulase and xylanase hydrolysis combined with carboxymethylation, crosslinking or acetylation showed higher SDF content, water expansion and retention abilities, and viscosity, resulting from the more porous and fragmented microstructure, larger surface area, and the introduction of more hydrophilic groups. MBDF, MBDF-HDEC, MBDF-HDEA, and MBDF-HDEPC all made the microstructure of H-EWG denser and more granular and decreased its α-helix content. Compared with MBDF, MBDF-HDEC, MBDF-HDEA, and MBDF-HDEPC more effectively increased the WRA, pH, hardness, chewiness, and gumminess of H-EWPG, and improved the gastric stability at 3–5 g/100 g. MBDF-HDEC, MBDF-HDEA, and MBDF-HDEPC were more effective to increase the antioxidant activity of the gastric and intestinal hydrolysates of H-EWG than MBDF (*p* < 0.05). These results highlight that the addition of MBDFs improved the microstructure, texture quality, digestion stability, and antioxidant activity of H-EWG, thus expanding its applications in food or other industries. Moreover, heating, cellulase and xylanase hydrolysis separately combined with carboxymethylation, acetylation and crosslinking are all simple, feasible, and environmentally friendly modification methods, and have potential commercial viability. However, this study just investigated the effects of the modified MBDFs on the properties of H-EWG; the action mechanisms of these composite modification methods impacting the properties of MBDF and egg white protein gels, and the stability of the modified gels need further work.

## Figures and Tables

**Table 1:** Effects of different composite modifications on the chemical composition, color, particle size, and hydration properties of MBDFs.

Chemical Composition	MBDF	MBDF-HDEC	MBDF-HDEPC	MBDF-HDEA
Moisture (g/100 g)	2.86 ± 0.21 c (10.84%)	2.54 ± 0.16 c (6.30%)	2.41 ± 0.07 c (2.90%)	2.94 ± 0.17 c (5.78%)
Fat (g/100 g)	2.47 ± 0.27 c (10.93%)	1.73 ± 0.07 c (4.05%)	1.24 ± 0.08 c (6.45%)	2.08 ± 0.10 c (4.81%)
Protein (g/100 g)	2.21 ± 0.08 c (3.62%)	2.07 ± 0.05 c (2.42%)	1.35 ± 0.11 c (8.15%)	1.48 ± 0.01 c (0.68%)
Ash (g/100 g)	3.07 ± 0.15 d (4.89%)	4.92 ± 0.47 c (9.55%)	3.40 ± 0.12 cd (3.53%)	4.36 ± 0.11 c (2.52%)
TDF (g/100 g)	88.95 ± 2.06 c (2.32%)	88.74 ± 1.76 c (1.98%)	88.60 ± 0.90 c (1.02%)	88.14 ± 2.08 c (2.36%)
SDF (g/100 g)	0.95 ± 0.09 e (9.47%)	5.28 ± 0.41 c (7.77%)	2.59 ± 0.11 d (4.25%)	2.07 ± 0.27 d (13.04%)
IDF (g/100 g)	88.09 ± 2.09 c (2.37%)	83.46 ± 1.46 e (1.75%)	86.01 ± 0.85 d (0.99%)	86.07 ± 1.84 d (2.14%)
Hemicellulose (g/100 g)	57.00 ± 6.23 c (10.93%)	44.34 ± 2.85 d (6.43%)	40.19 ± 3.13 d (7.79%)	45.44 ± 3.37 d (7.42%)
Cellulose (g/100 g)	19.56 ± 1.44 c (7.36%)	15.46 ± 1.84 d (11.90%)	14.26 ± 0.55 d (3.86%)	13.48 ± 0.76 d (5.64%)
Lignin (g/100 g)	11.53 ± 0.38 c (3.30%)	10.09 ± 0.52 c (5.15%)	9.75 ± 0.48 c (4.92%)	9.74 ± 0.82 c (0.84%)
*L*	75.22 ± 1.19 c (1.58%)	70.82 ± 2.42 d (3.42%)	66.69 ± 0.82 e (1.23%)	67.18 ± 1.01 e (1.50%)
*a*	8.34 ± 0.30 d (3.60%)	9.84 ± 1.06 c (10.77%)	9.50 ± 0.27 c (2.84%)	8.73 ± 0.42 cd (4.81%)
*b*	16.00 ± 0.63 d (3.94%)	19.20 ± 1.99 c (10.36%)	16.67 ± 1.13 d (6.78%)	14.91 ± 0.86 d (5.77%)
∆*E*	Control	6.56 ± 0.33 d (5.03%)	8.89 ± 0.62 c (6.97%)	8.13 ± 0.40 c (4.92%)
D_(3,2)_ (μm)	21.58 ± 2.36 c (10.94%)	19.27 ± 0.45 d (2.34%)	19.11 ± 1.01 d (5.29%)	18.59 ± 0.44 d (2.37%)
Polydispersity index	2.17 ± 0.06 c (2.76%)	1.81 ± 0.11 c (6.08%)	1.94 ± 0.06 c (3.09%)	0.84 ± 0.07 d (8.33%)
Surface area (cm^2^/cm^3^)	2768.63 ± 7.59 c (0.27%)	3112.79 ± 63.3 d (2.03%)	3138.17 ± 37.6 d (1.20%)	3226.70 ± 9.63 e (0.30%)
Water-holding ability (g/g)	1.79 ± 0.02 e (1.12%)	3.07 ± 0.12 c (3.91%)	2.35 ± 0.21 d (8.94%)	2.38 ± 0.10 d (4.20%)
Water-swelling ability (mL/g)	0.325 ± 0.010 e (3.08%)	1.03 ± 0.08 c (7.77%)	0.61 ± 0.07 d (11.48%)	0.82 ± 0.08 cd (9.76%)
Viscosity (cP)	12.33 ± 0.38 d (3.74%)	20.00 ± 3.00 c (15.00%)	18.33 ± 1.53 c (8.35%)	12.33 ± 1.52 d (12.33%)

Data are expressed as mean ± standard deviation (coefficient of variation). MBDF, millet bran dietary fiber; MBDF-HDEC, MBDF modified by heating and dual enzymolysis united with carboxymethylation; MBDF-HDEPC, MBDF modified by heating and dual enzymolysis united with phosphate crosslinking; MBDF-HDEA, MBDF modified by heating and dual enzymolysis united with acetylation; TDF, total dietary fiber; SDF, soluble dietary fiber; IDF, insoluble dietary fiber. Different lowercase letters (c–e) in the same row indicate significant difference (*p* < 0.05).

**Table 2:** Effects of different addition amounts of MBDFs on the texture properties of heat-induced egg white protein gel.

Gels	Amount (g/100 g)	Hardness (g)	Springiness	Cohesiveness	Gumminess	Chewiness (g)	Resilience
H-EWG		134.03 ± 3.52 f	0.90 ± 0.01 a	0.48 ± 0.04 a	63.92 ± 3.86 g	57.97 ± 2.89 f	0.04 ± 0.00 a
H-EWG/MBDF	1	139.93 ± 13.85 f	0.90 ± 0.02 a	0.49 ± 0.04 a	68.03 ±1.75 fg	61.33 ± 0.91 ef	0.05 ± 0.00 a
	2	166.80 ± 15.60 ef	0.88 ± 0.01 a	0.44 ± 0.01 ab	73.33 ± 5.03 f	64.69 ± 3.66 e	0.04 ± 0.00 a
	3	216.68 ± 19.16 cd	0.88 ± 0.01 a	0.44 ± 0.01 ab	95.28 ± 6.03 cd	84.70 ± 4.38 bc	0.04 ± 0.00 a
	4	245.49 ± 17.67 c	0.81 ± 0.03 b	0.41 ± 0.03 b	101.43 ± 6.12 c	82.36 ± 6.71 bc	0.04 ± 0.00 a
	5	258.50 ± 21.90 bc	0.86 ± 0.01 a	0.43 ± 0.03 b	111.88 ± 10.47 b	97.48 ± 16.61 a	0.04 ± 0.00 a
H-EWG/MBDF-HDEC	1	171.19 ± 12.82 e	0.88 ± 0.02 a	0.45 ± 0.06 ab	76.80 ± 4.04 f	67.69± 5.47 de	0.04 ± 0.00 a
	2	238.80 ± 19.07 c	0.82 ± 0.03 b	0.41 ± 0.02 b	97.13 ± 4.00 c	80.09 ± 2.96 c	0.04 ± 0.00 a
	3	203.43 ± 11.86 d	0.71 ± 0.16 c	0.44 ± 0.04 ab	89.60 ± 7.15 d	64.84± 19.25 e	0.05 ± 0.02 a
	4	263.90 ± 19.86 bc	0.81 ± 0.04 b	0.42 ± 0.02 b	111.55 ± 7.04 b	90.64 ± 1.69 b	0.04 ± 0.00 a
	5	323.69 ± 49.88 a	0.78 ± 0.02 b	0.39 ± 0.01 b	125.79 ± 15.71 a	98.87 ± 15.63 a	0.05 ± 0.01 a
H-EWG/MBDF-HDEPC	1	143.89 ± 5.88 f	0.87 ± 0.00 a	0.50 ± 0.03 a	71.34 ± 3.57 fg	62.66 ± 2.82 e	0.04 ± 0.00 a
	2	147.68 ± 12.67 f	0.87 ± 0.02 a	0.48 ± 0.04 a	70.49 ± 2.61 fg	61.98 ± 3.02 ef	0.04 ± 0.00 a
	3	184.43 ± 13.81 e	0.87 ± 0.04 a	0.49 ± 0.01 a	74.63 ± 6.21 f	79.59 ± 4.61 c	0.05 ± 0.00 a
	4	164.05 ± 22.50 ef	0.87 ± 0.03 a	0.46 ± 0.02 ab	90.75 ± 4.64 d	65.23 ± 2.55 e	0.04 ± 0.00 a
	5	272.20 ± 27.59 b	0.74 ± 0.15 bc	0.35 ± 0.06 b	97.11 ± 25.91 c	74.36 ± 4.92 cd	0.04 ± 0.00 a
H-EWG/MBDF-HDEA	1	181.79 ± 5.24 e	0.85 ± 0.04 a	0.41 ± 0.04 b	74.77 ± 8.52 f	63.81 ± 10.04 e	0.04 ± 0.00 a
	2	223.71 ± 8.97 cd	0.89 ± 0.04 a	0.44 ± 0.04 ab	83.23 ± 6.70 e	84.56 ± 9.67 bc	0.04 ± 0.00 a
	3	181.84 ± 11.92 e	0.86 ± 0.01 a	0.46 ± 0.03 ab	84.31 ± 3.85 de	71.62 ± 7.09 d	0.04 ± 0.00 a
	4	184.18 ± 12.30 e	0.87 ± 0.00 a	0.46 ± 0.01 ab	84.87 ± 7.69 de	73.89 ± 4.03 d	0.04 ± 0.00 a
	5	187.71 ± 13.18 e	0.88 ± 0.02 a	0.45 ± 0.02 ab	95.69 ± 27.33 c	75.39 ± 5.38 cd	0.04 ± 0.00 a

Different small letters (a–g) in the same column indicate significant difference (*p* < 0.05).

## Data Availability

The original contributions presented in the study are included in the article, further inquiries can be directed to the corresponding author/s.
